# “It Takes a Village”: Reflections from participants after a Hispanic community-based health promotion program

**DOI:** 10.1186/s12889-024-17737-1

**Published:** 2024-01-20

**Authors:** Bethany Korom, Meghan Malloy, Caroline Remmers, Elizabeth Welsch, Mari Cevilla, Zecilia Alamillo-Roman, Daniela Torres, Kelly Dione, David Nelson

**Affiliations:** 1 Medical College of Wisconsin, Milwaukee, WI USA; 2 United Community Center, Milwaukee, WI USA; 3 University of WI – Madison, Madison, WI USA; 4 Wauwatosa, USA

**Keywords:** Hispanic, Community-based intervention, Dynamic model, Nutrition & physical activity, Long-term follow-up

## Abstract

**Background:**

Physical activity (PA) among Hispanic and other minority adolescents in the U.S. lag behind White, non-Hispanic adolescents. Previous studies have examined the beneficial impact of culturally informed, community-based health programs within the Hispanic community on PA levels. There is a need for longer term follow up to determine the impact on family and individual habits over time. Our study aims to explore the aspects of a two-year PA and nutrition program, Families Inspired Together 4 Youth Empowered to Succeed (FIT4YES), that continue to influence family health habits and child development.

**Methods:**

Community-based focus groups were held in Milwaukee, WI with Hispanic parent participants of the FIT4YES program three years after program conclusion. A semi-structured guide of open-ended questions was used to facilitate the discussion of the lasting impact of FIT4YES. Each group was audio recorded, transcribed, and translated from Spanish when necessary. Four student researchers utilized a grounded theory qualitative approach to identify overarching themes.

**Results:**

Three focus groups with 16 total parents (*N* = 16) spoke about the program. Three overarching themes emerged from the transcripts indicating that cultural exposure, relationships, and self-growth were necessary for families to sustain the healthy behaviors promoted in FIT4YES. Specifically, parents discussed increased comfort levels with their children participating in school sports, the impact of shared experiences with community members, and continued skills initially taught and practiced during active programming.

**Conclusions:**

Our group adapted our previously published model to a “post-program” state that incorporated the major themes and sub-themes with levels of the social-ecological model. Although the FIT4YES program ended, multiple ideals instilled by the program continued, we believe, due to the common themes illustrated by our model. This study utilized a community check-in approach to gain insight into the long-term impacts of the FIT4YES program. We propose three recommendations for consideration in the creation of community-based health programs: utilize dynamic, culturally appropriate components based on the intended community; understand the strength of the program as a whole is dependent on the strength of each individual component; and incorporate an anchor institution for consistency and trust within the community.

**Supplementary Information:**

The online version contains supplementary material available at 10.1186/s12889-024-17737-1.

## Background

In the United States (U.S.) in 2022, the World Health Organization reported that 81% of adolescents aged 11–17 do not meet the 60 min of daily physical activity (PA) recommendations [1]. Similarly, in 2021, the CDC published results from the Youth Risk Behavior Survey identifying only 24% of high school students in the U.S. reported being physically active for at least 60 min per day in the seven days prior to the survey [2]. The benefits of a healthy lifestyle including PA and adequate nutrition for the prevention of chronic disease development are well established; however, PA levels among Hispanic and other minority adolescents in the U.S. continues to lag White, non-Hispanic adolescents (20% of Hispanic adolescents compared to 25% of non-Hispanic White adolescents in grades 9–12 report achieving 60 min or more of PA daily) [3], a factor likely contributing to the continued differences in childhood obesity, with 25% Hispanic adolescents meeting the qualifications for obesity in 2018 (BMI greater than or equal to age- and sex-specific 95th percentile of CDC growth charts) compared with 16% of non-Hispanic White adolescents [4]. Many programs that challenge this disparity often fail to address the social determinants of health, including the environmental and behavioral factors that influence individual and community well-being, introducing a role for integrating program development within the context of the social-ecological model (SEM) [5, 6]. However, programs with only behavior-oriented interventions (individual-based, such as individual education) have demonstrated limited long-term effects, emphasizing an integrated program with community-/environment based efforts for sustainable results [7].

Previous studies have examined the beneficial impact of culturally informed, community-based health programs within the Hispanic community on nutrition expertise and exercise levels and promote the development of health promotion programs that are culturally aware in both “surface structure” (language, settings, materials) and “deep structure” (social, environmental, psychological) components [8]. These programs acknowledge the importance of including cultural structures and practices, reinforcing the benefits of involving the family and social support, having literacy-level appropriateness, and utilizing cultural values to promote long-term behavior change [9]. When designing behavioral interventions for Hispanics, one must be aware of the diversity within the Hispanic population and can utilize formative research to tailor an intervention to a specific community, and must be willing to adjust delivery agents, materials, literacy levels, and support based on the population’s central values and behaviors [9]. There is a need for a more in-depth look at the components of these community-based health programs that lead to sustained behavior change within the individual, community, and societal levels to create lasting change within communities at risk for poor health outcomes.

In a recent paper published by our team [10], we describe a community-based health promotion program, Families Inspired Together 4 Youth Empowered to Succeed (FIT4YES), and the immediate effects on family members following the two-year program (2018–2020). Briefly, FIT4YES was created with community-based partnerships between the United Community Center (UCC), Marquette University (MU), the Medical College of Wisconsin (MCW), and a community participant advocate (parent). The program invited 50 Hispanic students (ages 10–11) considered overweight and their families to participate in combined gender after-school activities, educational sessions, and family-centered retreats surrounding PA and nutrition education. FIT4YES was delivered four days per week with additional adult and family participation times each month at the UCC and the connecting middle school on the south side of Milwaukee, Wisconsin. FIT4YES introduced multiple new sports and activities including mountain biking, snowboarding, and camping, while providing transportation and equipment to borrow to assist with financial and logistical barriers. The program held nutrition sessions to teach family participants new cooking skills to make healthy, culturally inspired recipes. FIT4YES hosted parent meetings to support the growing community of parents and allow them to connect and grow together on their health journey.

Immediately after the program concluded in 2020, 24 interviews with parent and student participants were held via Zoom to discuss how the program impacted family routines and health awareness. Based on the initial program evaluation using a qualitative, grounded theory approach, our team created a novel model informed by the SEM that describes the aspects of the FIT4YES program that allowed parents and families to embrace trying new and healthy activities. We used common themes that emerged from the family interviews after the program to determine the vital components that led to the success of the program, evidenced by positive behavior change discussions during Zoom interviews. Three overarching themes describe the key components of the FIT4YES program: strength in relationships, engagement with entire families, and the interplay of common subthemes [10]. A community-engaged practice of gaining feedback at all points of the research would add value to the validity of the project and research to date.

To advance health equity within public health, design thinking has been proposed as an innovative theory to incorporate feedback from the population to integrate the subjective and dynamic interpretations of health in programs or projects, producing increasingly successful iterations [11]. Utilizing population feedback in this way can lead to more success in the long-term for health-promotion programs in a specific population. Triangulation is a process of obtaining and demonstrating how information included in a study connects with each other and is often utilized to enhance the study’s validity and dependability of the data despite the narrative text’s thickness and richness [12, 13]. Interventions can integrate triangulation into steps from initial program design to post-conclusion evaluation by connecting back with a community advisor for input. Despite having a community member on our initial team of the FIT4YES program to support the cultural analysis, it was deemed important to return to the participants themselves to determine the relevancy of the program in the present. Immediate health promotion program results may not predict long-term effects on family health status.

Despite the plethora of studies examining the impact of health-promotion programs within the Hispanic community, there is an absence of longer-term follow-up supported by the literature [14, 15]. Many programs report their outcomes immediately following the conclusion of the intervention due to limited resources and ultimate funding restraints, like the qualitative interviews following the FIT4YES program, but results beyond a year out are rarely described [16]. Longer-term follow-up is beneficial to explore factors leading to sustained behavior change to continue to incorporate or modify in future programming. This study aims to explore the aspects of FIT4YES that continue influence family health habits and child development three years after active program conclusion.

## Methods

Using a qualitative research approach, three focus groups were held with parents who participated in the two-year FIT4YES program to explore the program’s impact on family health habits and their children's development three years after active program conclusion. Focus groups were led by three medical student interviewers and the overseeing PI at the community partner site (3 focus groups with 16 total participants). Each focus group averaged 60 min (Range 53–65 min). The study was approved by the MCW IRB committee before recruitment.

### Recruitment

Participants in this study were invited to participate if they had previously participated in the FIT4YES program. Participants were contacted by the previous coordinator of the FIT4YES program via phone, in-person conversations, and follow-up text messages to gauge interest in attending focus group sessions. Three dates were proposed in February and March of 2023. Parents received an informational letter, had an opportunity to ask questions, and gave verbal informed consent to participate before data collection as approved by the Medical College of Wisconsin Institutional Review Board committee. Children were invited to join their parents for food and beverages to accommodate for after-school care; however, no data were collected from children.

### Data collection

A semi-structured guide of open-ended questions was used to facilitate the focus group discussions. Questions and guides were not provided ahead of time and were not pilot tested. Questions were approved by the native Spanish speaker for cultural sensitivity. The question guide developed for this study is provided as Additional file 1. Three of four female medical students led the focus groups (BK, MM, CR, EW) with the male senior mentor (DN, PhD) observing and asking additional questions when appropriate. DN is a professor in the department of family and community medicine at MCW and has advanced training in community-based participatory research in a variety of settings. He understands how to work with community members and organizations and has acted as a mentor and trained medical students BK, MM, CR, and EW in open-ended interviewing. All researchers had worked extensively with the UCC prior to study commencement, and DN led the initial participant interviews along with translator and author MC, described in our prior study [10]. All participants were aware of the reasons for the research, were given a letter of consent, and volunteered to take part in the interview process.

Parent focus groups took place within the UCC from 5:00 PM to 6:30 PM to accommodate for the busy schedules of parents, and participants received food and beverages to promote a casual environment to talk with our researchers. Each participant’s involvement lasted approximately 1.5 h (60 min of recorded discussion with 30 min built in to arrive, connect, and eat), and each participant was compensated with a $25 VISA gift card after the focus group. No repeat interviews were conducted. After three focus group meetings and inclusion of all interested participants, repeat themes were evident and data saturation was discussed.

While the general discussion questions were predetermined, when common themes were presented, additional follow-up questions were asked for more clarification. The discussion focused on how the FIT4YES program continues to influence their families and children a few years after the program and why they believe certain aspects were impactful. Participants were also shown the model developed from previous interviews and asked for feedback.

### Data analysis

Each focus group was audio recorded using ‘Otter.ai,’ and transcriptions were stored on a secure Box platform. Some participants preferred to answer the discussion questions in Spanish. The audio recording was transcribed verbatim. Fluent Spanish speakers on the research team translated the necessary Spanish responses into English, paraphrasing as needed while keeping the overall message consistent. Some responses were excluded due to the poor quality of the audio recording. Transcripts were not returned to participants for comments or corrections. Field notes and bracketed comments were made and talked about during the interview.

Four medical student researchers (BK, MM, CR, EW) individually analyzed the transcripts using grounded theory inductive qualitative analysis under the supervision of the PI (DN) [17]. This process was similar to the one used by some of the investigators in previous studies [10, 18, 19]. The methodology used was consistent with our previous project analyzing the FIT4YES program. Before the start of this analysis, three of the medical student researchers (BK, MM, CR) trained the fourth student (EW) on grounded theory qualitative analysis in the manner that the PI had previously trained them. The team utilized an open coding and constant comparison approach to develop the initial coding structure based on themes that emerged from the transcripts of all three focus groups. After coding each transcript, the researchers met weekly to discuss emerging themes and resolve individual differences. When all three transcripts were coded, the medical students and senior mentor met to discuss the initial coding structure and ultimately consolidated eight initial themes into three overarching themes. The team collaboratively developed definitions for each theme and provided examples to support the identified phenomenon utilizing text from interview transcripts. The theme, definition, and text examples make up the codebook for this analysis. Inter-coder reliability was attained via weekly discussions until an agreement was met. In our previous project, a novel version of the SEM was developed, intended to be dynamic and readily adaptable to new findings. Team members met to consider how the previous model needed to be modified to reflect the newly identified themes, leading to the creation of an updated iteration of our model. The investigators met with the UCC to present the updated model and codebook findings for feedback and open discussion, and the model and codebook definitions were updated accordingly. COREQ checklist for qualitative research is provided as Additional file 2.

## Results

Twenty-three parents were contacted, and 16 total parents attended one of the three proposed dates (total *n* = 16, 69% response rate, each individual date *n* = 6, 7, 3 parents respectively). Interview participants represented 15 different family units, with 15 female (mother) participants and one male (father) participant present at only one of the three focus groups. Collaborative analysis of the three session transcripts allowed the identification of three major themes demonstrating what participants believed made this program successful. Repeated themes included cultural exposure, relationships, and self-growth. Additional file 3 depicts quotes that illustrate each of the three themes and associated sub-themes. These themes are consistent with the data presented and the findings.

### Cultural exposure

Cultural exposure is defined as the interaction of a specific community, in this instance, a Hispanic community on the south side of Milwaukee, WI, with thought patterns that expand their unique cultural norms including healthy activities derived from traditionally mainstream “American” experiences. This includes the subthemes of *accessible resources* and the *United Community Center.* Throughout the interviews, participants often commented on their personal upbringing within their Hispanic culture and the subsequent challenges of raising their children under the umbrella of American culture. The FIT4YES program at the UCC was a safe environment to explore sports, outdoor activities, and food preparation methods unfamiliar to parents and children. Multiple parents commented on feeling more comfortable letting their children participate in sports at school like swimming and snowboarding and organizing camping trips for their families—experiences the parents were not exposed to growing up.

*“We haven’t had the opportunity to give our kids the toys or teach them the sports that are completely simple and normal for you guys [Americans] that aren’t for us [Hispanics]. We haven’t seen a lot of this before, at least I haven’t. I feel like you gave us the opportunity to know what sports are like in the American culture which I believe is very important” – Parent 6, Group 1*

### Relationships

We defined relationships as the interpersonal connections between family and community members that grow through shared experiences and common goals. Two subthemes were identified as *family cohesion* and *community interconnectedness* to further elaborate on the sentiments expressed by interview participants. In both cases, relationship development was expanded through performing challenging activities together and through the introduction of new ideas to the group. Many parents talked about the relationships from the program that have continued to influence their children’s lives, from mentors and teachers to friendships with peers and siblings. The common sentiment of “It takes a village to raise a child” was introduced during the program as parents became more comfortable relying on each other with their children.

*“One of the meetings we initially had with Dr. Paula, she said “it takes a village to raise a child”. And I never had heard of that term... I was like, what does she mean? And she explained it, about how even though I'm not the parent of this child, we're a community and we look out for each other's children. And I guess it didn't make sense until we were all sitting together at a campground. And you're not only looking to make sure your kids are okay, counting your children, but you're making sure they're all here. Then it made sense, you know, like wow, okay, I get it” – Parent 1, Group 3*

### Self-growth

We defined self-growth after the FIT4YES program as the sustained motivation to engage in healthy behaviors. This includes the subthemes of *acquiring new skills* and *self-reflection* to promote healthy habits. Many families discussed barriers previously preventing them from certain activities, specifically the required high price or uncommon materials. Even so, parents brought up the specific experiences and skillsets that the program promoted and they themselves chose to continue including continued meal planning using the provided InstaPot, family bike ride excursions, and, in the winter months, choosing to purchase memberships to indoor jumping gyms to stay active. Many parents commented on the new sports their children started to play competitively in high school because of the exposure and practice they received in the FIT4YES program.

*“I think a lot of times we stick to what we know. So, for my son, we would have just done the MPS [Milwaukee Public School] $36 swimming lesson, you know, level one, level two, but because of this [FIT4YES], he was able to take swimming lessons at Augustine prep, and right now he's on his second year at Marquette High School. He's on the swim team.” – Parent 2, Group 1*

*“They [FIT4YES] made me make the space and time on my schedule to do the activities ... that made you notice that you can do it. And you have to find the space to do those activities ...So yeah, I learned that putting those things in your schedule is good for your family. And it's good for yourself” – Parent 2, Group 3*

Overall, the FIT4YES program had a lasting impact on its participants as demonstrated by their engagement and continued activities three years later.

## Discussion

Community-based health promotion programs impact the attitudes and behaviors of individuals while programming is taking place, but their influence does not end there. The experiences that participants have affect their health and well-being for years after the conclusion of the program. However, the literature often lacks long-term follow-up with communities, making it difficult to understand programs’ lasting impacts on communities. In this project, we returned to the community to explore the aspects of the FIT4YES PA and nutrition program that continued to influence participants three years later, and discussed possible mechanisms that allowed for the continuation of these behaviors.

Based on our analysis of the interviews, we found that cultural exposure, relationships, and self-growth were necessary ingredients for long-lasting change within this community. The literature also acknowledges the importance of addressing “deep-structure” cultural characteristics such as the values, traditions, acculturation levels, and psychological influences within a community when developing culturally appropriate programs, rather than focusing on only superficial cultural characteristics such as language [9, 20–22]. The importance of relationships is also commonly described in the literature. Within the literature, “familism” is an important value frequently studied within the Hispanic community leading to the promotion of health and wellness [23] and has been recommended to be included in the creation of community-based programs [8, 9]. Family dynamics have previously been shown to strongly influence individual nutritional choices. Specifically, within the Hispanic community, social support has been identified as the strongest predictor of PA [6, 24, 25]. Safe and trusting relationships between participants and program leaders increase the likelihood of community members engaging in new behaviors [26, 27]. Similarly, exposure to challenging experiences within the safe environment of a community program allows students to develop new skills within that curriculum but also enhances student self-efficacy to engage in novel, challenging activities outside of the context of the program [28, 29]. The FIT4YES program was initially developed based on these values to create culturally appropriate programming, utilizing long-standing community partnerships, prior iterations of the program, and community member advocates to identify the most effective intervention tactics for the families within this specific community. This program was built upon a strong-rooted trust within the community that was previously established through the familiar faces of the programming staff and combination of three partner institutions within the community.

From our team’s initial qualitative analysis of family interviews immediately after the FIT4YES program, themes reflected the dynamic nature of the program characteristics, and a model emerged based on an adaptation of the SEM. We initially reflected on the static nature of the widely accepted SEM and discussed how this model cannot accurately illustrate how aspects within a program synergize through dynamic interactions at all levels. Our model integrated the derived themes of FIT4YES with the levels of the SEM, linking program “ingredients” into a funnel, which ultimately results in the empowerment of youth to engage in new and healthy activities. This iteration of our program-specific model was intended to be dynamic and evolve over time with new knowledge and experiences. The first model, with revisions after community partner feedback, is provided in Additional file 4.

**Fig. 1:**
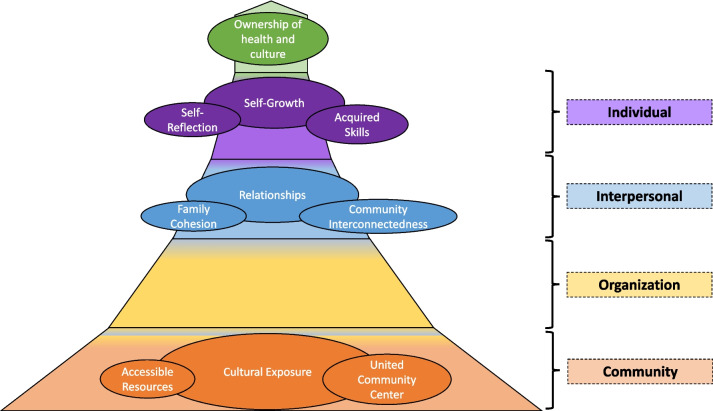
“It takes a village:” Community-Based Health Promotion Model. This model integrates the SEM levels with the three overarching themes (Cultural Exposure, Relationships, Self-Growth) and associated sub-themes that emerged from the analysis of the focus group interviews when discussing the lasting impact of the FIT4YES program on family behaviors. Each theme is built on top of each other to build towards the highest level: Ownership of health and culture. Each level’s colors blend to demonstrate the flexibility and integration of each level with the next

The themes from our current work represent new information gleaned from parent interviews about the long-term impacts of a health promotion program, necessitating the revision of the previous model to reflect these experiences. After narrowing down common themes to three overarching topics with two sub-themes each, an updated model emerged to represent the change in behaviors over time, as shown in Fig. 1. It must be noted that this is not intended to be a final iteration of the model, as all individuals, communities, programs, and organizations change over time. Instead, this model can be adapted indefinitely to fit the needs of new communities, individuals, ideas, and times.

The absence of comments that fit within the organization level became increasingly apparent with each focus group. Some parents commented on the difficulty of engaging their children in activities without the formality of a structured commitment with mentors and facilitators outside the family unit, reinforcing a common experience. Existing literature supports the beneficial effect on weight management of culturally tailored programs that program leaders directly supervise for the duration of the program [30, 31]. Even though the group consensus favored the reimplementation of the FIT4YES program, multiple ideals instilled by the program remained intact, inspiring the continuation of healthy substitutions into meal-time recipes, activities around the neighborhood, and choosing to become more health-aware throughout daily life. Particularly, our “self-reflection” theme under the self-growth category incorporates each participant’s individual choices. The new skills and knowledge that were gained throughout the program act as an additional tool for families to support health. Not every aspect of the program continued for each family, but the ability for families to reflect and decide for themselves what matters most, we believe, has helped with sustained behavior changes over time.

**Fig. 2:**
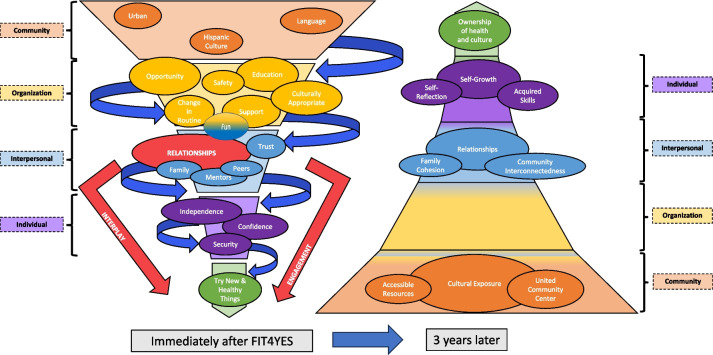
Model Evolution Over Time. Our models continue to incorporate themes from the interviews into the levels of the SEM. During the FIT4YES program, the organization utilized the common community levels to funnel important ingredients down to the individual. After the program, the common community, memories, and relationships act as the base to hold the individual up to promote ownership of their own health and culture. This is supported by the skills and self-reflection within the interpersonal level, allowing individual ownership of decisions and leading toward sustained behavior change. Confirmation of the new model also demonstrates the necessity of community-based organizations tapping into the culture of a community for a lasting impact

Our previous model utilized the community and organization to build a safe environment and provide opportunities to gain new skills and experiences. Without the program intentionally creating this top-down “flow,” the community becomes the anchor for sustained change. The individual level is now at the top, supported by each of the levels below. The program ultimately created a strong foundation to continue through the three themes: cultural exposure, relationships, and self-growth. It left behind a legacy with strong memories that continue to influence participants’ decisions. The effect is similar to casting a shadow throughout the community because it occurred within a common community center, the UCC, leading to a constant reminder of the shared experiences with each passing. The ease of remembrance and longing for a new program demonstrates the deep-rooted seeds planted within each individual family. The model progression is shown in Fig. 2.

While it is worthwhile to note that the conclusion of the FIT4YES program led to the loss of structure at an organizational standpoint, it is equally important to emphasize that even without its continued presence, many of the healthy behaviors and life lessons taken away from the program still stuck with the families over time. The children who participated in the program as middle schoolers are now graduating high school, and their parents are moving on to different phases of their lives. Additionally, the discontinuation of the program was compounded by additional life stressors because of the COVID-19 pandemic. Despite this change, our recent discussions with program participants have shown that, while they have evolved as families, their healthy behaviors have evolved with them, even without the accountability provided by a structured program.

Our previous study discussed the interplay of the various components that made this program successful, such as the interpersonal relationships that created a safe, culturally appropriate environment for participants to try new healthy behaviors. This study validates these findings and demonstrates the complexity of creating a sustainable program within this Hispanic community. Perhaps it is not the strength of the program which determines its success. Rather it is the strength of its individual components that will ensure the longstanding impact of a community-facing health education program. Like building blocks when a tower is created with intention and a solid foundation, the structure remains intact even if one piece is removed. A successful community-engaged program depends on the strength of its parts, and the strength in what remains, despite the conclusion of formal programming, can help drive the evolution of a community towards a healthier future. These results may be specific to this Hispanic community, which shared a strong bond already due to its geographical location with a longstanding community center at its core; however, the value of the individual components of this ecosystem that make up the whole cannot be understated in implementation of future programs and should be explored in other communities.

The impact of the FIT4YES program was largely catalyzed by its integration within the UCC, a long-standing institution dedicated to the Hispanic community in Milwaukee. The UCC and MU created the FIT4YES program to embody the mission of the UCC: “to transform the lives of Hispanics, families and individuals of all ages by providing the highest quality comprehensive services in education, human services, health, community development and cultural arts” [32]. The harmony between the UCC’s standing commitment to its community and FIT4YES allowed for seamless integration of the program into the fabric of the community. It must be acknowledged that this foundational anchor may be difficult to replicate. The UCC has remained a constant for over 40 years in the Milwaukee community, allowing it to develop into the integral role it now plays. An organization so devoted to and beloved by the community it serves is difficult to recreate but is essential to the success of implementing a program such as FIT4YES in which longevity is a key outcome.

*“Because of this [FIT], we had the exposure to those activities, like how to do it, how to access it, and then also helping us financially... We were never going to take him skiing or snowboarding or rock climbing or mountain biking or camping. You know it after Fit that finally, we went camping for the first time like as a family, but if it wasn't for FIT none of that would have ever happened.” – Parent 2, Group 1*

## Conclusions

Based on our groups’ analysis of focus group interviews, we have compiled three recommendations based on the continued validation and evolution of our models:

1. Multiple components are needed for the success of a health and wellness program that must be dynamic in nature to meet the community’s needs in a culturally appropriate way.
2. The lasting strength of a community-based program is dependent on the strength of the individual components. These components will differ based on the individual organization and the community in which it is based.
3. An anchor institution is vital for a longstanding effect allowing consistency and trust within the community.

### Limitations

- Qualitative research cannot be generalized to other populations, but we believe the depth of focus groups provides general themes that can be relatable to other groups.
- While we did not interview every parent who had been included in the first round of interviews, our response rate of 69% represents a majority of the families that had previously participated.
- The quality of the audio recordings and transcription using ‘Otter.ai’ with responses in Spanish required the exclusion of some responses from our analysis. While the medical students who translated responses from Spanish to English are fluent, they are not native speakers and may have missed nuances of responses. However, we kept the overall message consistent and had native Spanish-speaking partners at the UCC available for clarification questions as necessary.
- Throughout our focus groups, we discussed the community member input on the evolution of our group’s model over time. All the feedback we received was positive and confirmed that the model was accurate. The main intent of the activity was to see what aspects from the program stayed with the families. Few program evaluations get to return after the funding has ended. The deep relationships between the community and academic partners facilitated the meetings and the additional evaluation. Since funding was not available to continue, despite family interest, the team did not want to give false hope that seeking suggestions would indicate the program was going to return.

### Public health implications

There are clear public health implications for this project and others that are to follow. First, long-term follow-up should be necessary for community engagement and evaluation. However, many community-engaged projects are grant-funded and thus time-limited. In addition, a strong commitment to community engagement means partnerships continue with or without funding [33]. The longstanding commitment to community organizations meant that further additions to the literature and practice are possible with very little additional investment. The project demonstrated the buy-in from the community. Multiple parents asked if the project was starting again as they saw and felt the benefit for their children, families, and community. When there is an ongoing interest and need, partnerships need to do their best to fulfill the wants and needs of the community.

### Supplementary Information

**Additional file 1. **Interview question guide. All questions were approved by a native Spanish speaker for cultural sensitivity. 

**Additional file 2. **COREQ checklist. In accordance with BMC Series editorial policies, our manuscript reporting adheres to COREQ guidelines.

**Additional file 3. **Participant quotes from focus groups. This table illustrates specific quotes from the focus groups that best demonstrate each respective theme and associated sub-themes that emerged from the qualitative analysis. 

**Additional file 4. **“It’s about being healthy:” Community Based Health Promotion Model. The creation of this novel model was based on themes that emerged from prior family interviews that allowed for program success. Our model integrates the levels of the SEM with aspects of the FIT4YES program that were necessary to empower participants to engage with healthy behaviors. Each of the factors included are linked in such a way to create a funnel effect down to the individual level allowing for change within the larger community.

## Data Availability

The data that support the findings of this study are available from the corresponding author upon request.

## Abbreviations

U.S: United States
PA: Physical activity
CDC: Centers for Disease Control and Prevention
SEM: Social-ecological model
FIT4YES (FIT): Families Inspired Together 4 Youth Empowered to Succeed
UCC: United Community Center
MU: Marquette University
MCW: Medical College of Wisconsin
IRB: Institutional Review Board
PI: Principal Investigator
