# Supplementary material for: “It Takes a Village”: Reflections from participants after a Hispanic community-based health promotion program
Source: BMC Public Health. 2024 Jan 20;24:237. doi: 10.1186/s12889-024-17737-1 (PMC10799519; doi:10.1186/s12889-024-17737-1)
Supplement: Supplementary file 1 — Additional file 1. Interview question guide. All questions were approved by a native Spanish speaker for cultural sensitivity. [file 12889_2024_17737_MOESM1_ESM.pdf]

Additional file 1: Interview question guide. All questions were approved by a native Spanish speaker for cultural sensitivity.

We will ask a similar variation of questions around the following topics: trying new things, health and wellness, and family cohesiveness. We will ask elaboration questions when necessary.

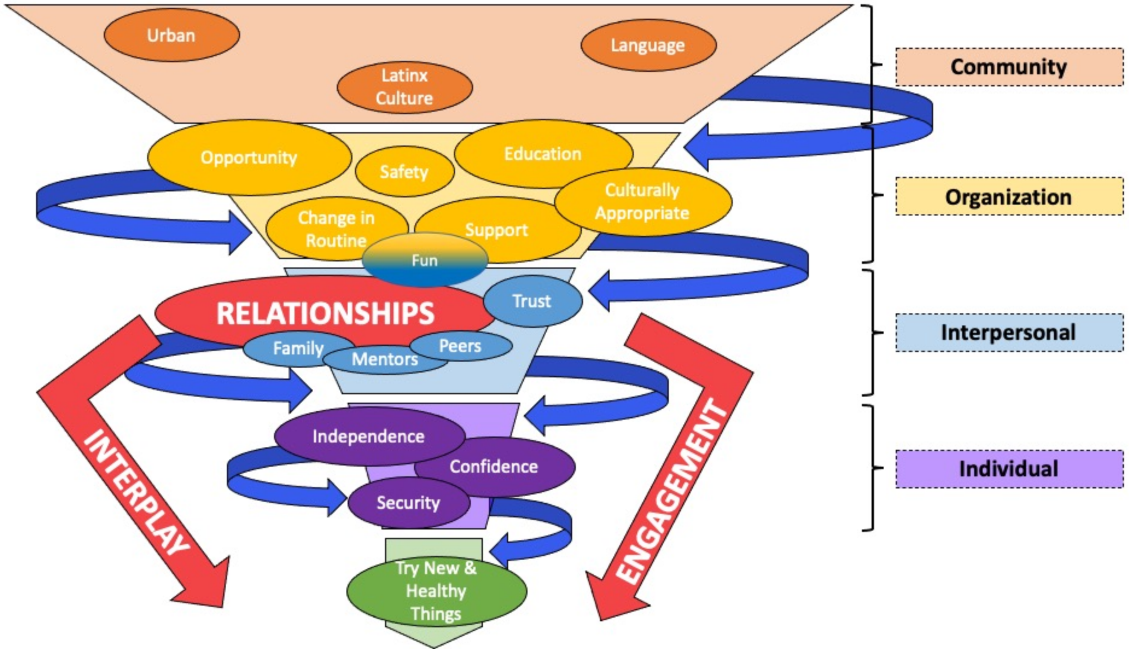

You all participated in a program, **FIT 4 YES**, two years ago.

1. How has the program influenced your family?

2. How has it influenced your health and wellness?

3. Tell me what you remember from the program.

4. From this model, what is most important for volunteers to know before getting involved with the community?
