# Supplementary material for: “It Takes a Village”: Reflections from participants after a Hispanic community-based health promotion program
Source: BMC Public Health. 2024 Jan 20;24:237. doi: 10.1186/s12889-024-17737-1 (PMC10799519; doi:10.1186/s12889-024-17737-1)
Supplement: Supplementary file 3 — Additional file 3. Participant quotes from focus groups. This table illustrates specific quotes from the focus groups that best demonstrate each respective theme and associated sub-themes that emerged from the qualitative analysis. [file 12889_2024_17737_MOESM3_ESM.pdf]

Additional file 3: Participant quotes from focus groups. This table illustrates specific quotes from the focus groups that best demonstrate each respective theme and associated sub-themes that emerged from the qualitative analysis.

Theme	Subthemes	Quote	Participant
Cultural Exposure	Accessible resources	“Helping us like financially. It was never like, an obstacle, you know, it's like money. We would have you know, pay for something a little bit but nothing like where it's gonna be like No, you know, where we can't do it because it's too much.”	Interview 1, Parent 2
	United Community Center	"What makes this program special is that it was held here at UCC and because the kids know each other from when they were in kindergarten, so they grew up together"	Interview 1, Parent 2
Relationships	Family cohesion	“I think that this brought to our family, it taught us a lot about unity, about team. And not only in our comfort zone, but we got a little bit out of our comfort zone. And it brought us closer as a family, we did a lot of things as a family, but with FIT4YES, we had a lot of fun, things that I probably wouldn't have planned on our own”	Interview 3, Parent 1
	Community interconnectedness	"We met many parents and our kids made very good friends and it was beautiful and we did life with many families that normally we would not have. And we hadn’t known each other but we could pass time together camping or whatever, but it was really nice to have that. And then my son made some really really good friends with the program. You know, kids maybe he wouldn't have talked to during the school day and they became really great friends participating in this"	Interview 2, Parent 6
Self-growth	Acquiring new skills	“My daughter didn’t know how to cook, or cut or hold a knife. She learned a lot in the kitchen and really liked it.”	Interview 1, Parent 5
	Self-reflection	“[FIT] made me make the space and time on my schedule to do the activities so that make a big difference. Because that make you notice that you can do it. And you have to find the space to do those activities. Because it was more the commitment was for doing something fun and beneficial directly for you not to go in in a meeting. It was in a meeting, there's going to give you more things. So yeah, I learned that putting those things in schedule is good for your family. And it's good for yourself”	Interview 3, Parent 2
