# Supplementary material for: “It Takes a Village”: Reflections from participants after a Hispanic community-based health promotion program
Source: BMC Public Health. 2024 Jan 20;24:237. doi: 10.1186/s12889-024-17737-1 (PMC10799519; doi:10.1186/s12889-024-17737-1)

Additional file 4: "It's about being healthy:" Community Based Health Promotion Model.

The creation of this novel model was based on themes that emerged from prior family interviews that allowed for program success. Our model integrates the levels of the SEM with aspects of the FIT4YES program that were necessary to empower participants to engage with healthy behaviors. Each of the factors included are linked in such a way to create a funnel effect down to the individual level allowing for change within the larger community.

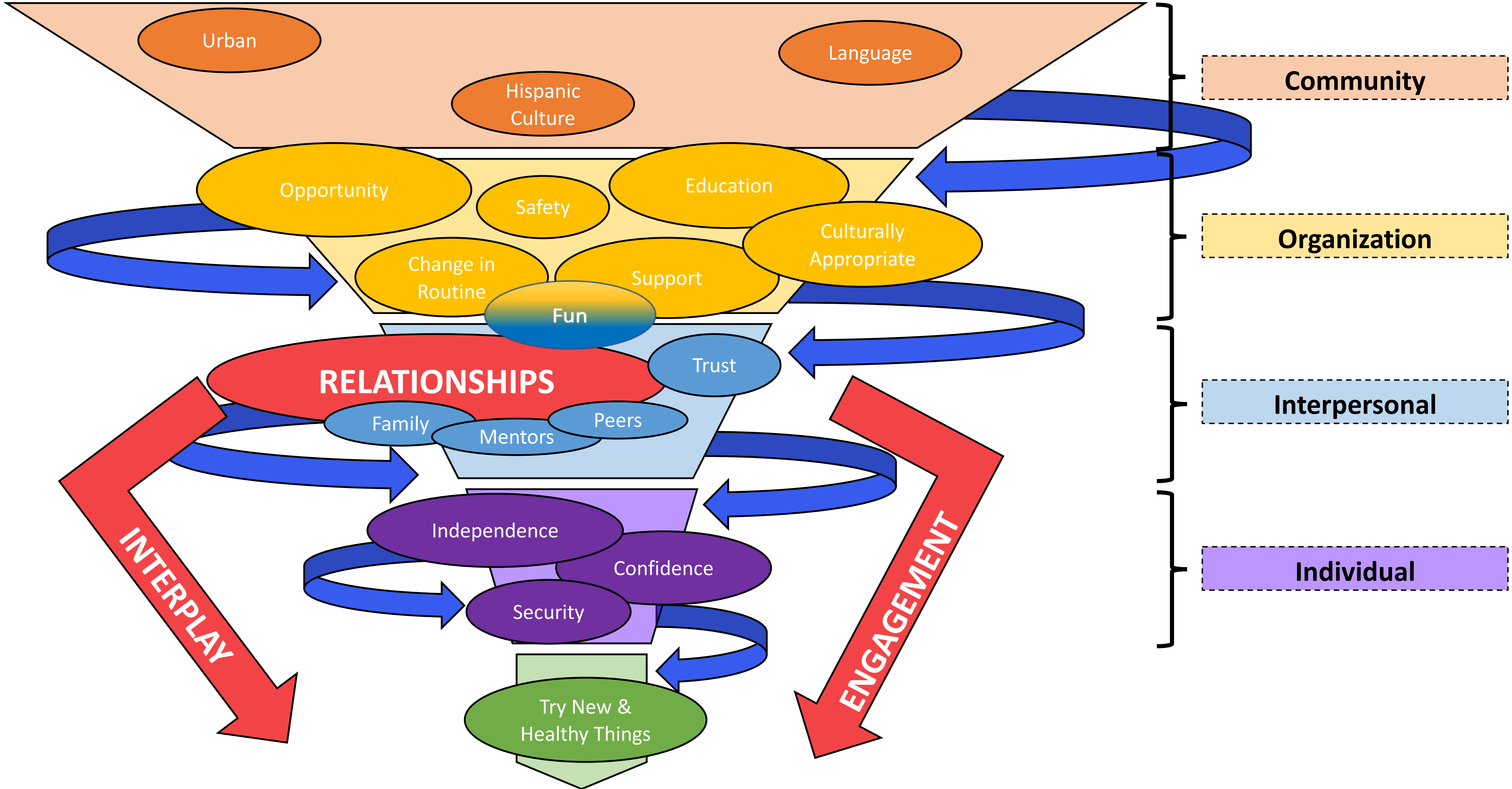

Supplement: Supplementary file 4 — Additional file 4. “It’s about being healthy:” Community Based Health Promotion Model. The creation of this novel model was based on themes that emerged from prior family interviews that allowed for program success. Our model integrates the levels of the SEM with aspects of the FIT4YES program that were necessary to empower participants to engage with healthy behaviors. Each of the factors included are linked in such a way to create a funnel effect down to the individual level allowing for change within the larger community. [file 12889_2024_17737_MOESM4_ESM.pdf]
